# Development of novel catalytically active polymer-metal-nanocomposites based on activated foams and textile fibers

**DOI:** 10.1186/1556-276X-8-238

**Published:** 2013-05-16

**Authors:** Berta Domènech, Kharla K Ziegler, Fernando Carrillo, Maria Muñoz, Dimitri N Muraviev, Jorge Macanás

**Affiliations:** 1 Department of Chemistry, Universitat Autònoma de Barcelona, UAB, Bellaterra, Barcelona, 08193, Spain; 2 Chemical Engineering Department, UPC, Universitat Politècnica de Catalunya, C/Colom, 1, Terrassa, Barcelona, 08222, Spain; 3 INTEXTER, Universitat Politècnica de Catalunya, UPC, C/Colom 15, Barcelona, 08222, Spain

**Keywords:** Metal nanoparticles, Polyurethane foams, Polyacrylonitrile fibers, Polyamide fibers

## Abstract

In this paper, we report the intermatrix synthesis of Ag nanoparticles in different polymeric matrices such as polyurethane foams and polyacrylonitrile or polyamide fibers. To apply this technique, the polymer must bear functional groups able to bind and retain the nanoparticle ion precursors while ions should diffuse through the matrix. Taking into account the nature of some of the chosen matrices, it was essential to try to activate the support material to obtain an acceptable value of ion exchange capacity. To evaluate the catalytic activity of the developed nanocomposites, a model catalytic reaction was carried out in batch experiments: the reduction of *p*-nitrophenol by sodium borohydride.

## Background

In the last decade, heterogeneous catalysts have attracted much interest because of their general advantages [1] that have been boosted thanks to the use of nanomaterials [2-4]. In fact, nanoparticles (NPs) are increasingly used in catalysis since their enhanced reactivity significantly reduces the quantity of catalytic material required to carry out reactions with a high turnover [1,2,5]. However, following the basic principles of nanosafety, the prevention of uncontrollable escape of these materials to the reaction media as well as the minimization of the probability of their appearance in the environment is becoming a crucial issue [3-6]. In this sense, the synthesis of polymer-metal nanocomposites (PMNCs) [1,7-10], obtained by the incorporation of metal nanoparticles (MNPs) in polymeric matrices, has demonstrated to be an attractive approach [5,8]. By stabilizing MNPs in a polymeric matrix, it is possible to prevent their escape to the reaction medium, thus providing an easy separation of the catalyst from the reaction mixture which, in turn, allows the possibility to reuse the catalytic species without losing efficiency.

One of the methodologies that allow obtaining these PMNCs in a feasible way is the so-called intermatrix synthesis (IMS) [8,11,12], based on the dual function of the matrix, which stabilizes the MNPs preventing their uncontrollable growth and aggregation and provides a medium for the synthesis. IMS proceeds by a simple two sequential steps: (a) the immobilization of metal cations (MNPs precursors) inside the matrix and (b) the reduction of metal ions to the zero-valent state leading to the formation of MNPs.

The main goal of this work is the development of advanced nanocomposite materials obtained by the incorporation of silver nanoparticles (AgNPs) in typical textile fibers (polyacrylonitrile, PAN, and polyamide, PA) and in polyurethane foams (PUFs). Yet, up to now, the IMS technique has been applied to polymers bearing ionogenic functional groups that retain the MNPs ion precursors [8,13,14]. Regarding this issue, and taking into account the nature of some of the polymeric matrices (e.g., PUF), it was considered essential to activate the support material to obtain an acceptable value of ion exchange capacity (IEC).

Finally, in order to evaluate the catalytic activity of the different developed PMNCs, a model catalytic reaction was carried out in batch experiments: the reduction of *p*-nitrophenol (4-np) to *p*-aminophenol (4-ap) in the presence of NaBH_4_ and metallic catalyst [15].

## Methods

### Materials

**Figure 1:**
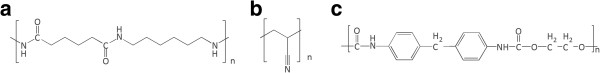
Structural units of the polymeric matrices (a) PA, (b) PAN, and (c) PUF.

Commercial PUF was obtained from Comercial del Caucho (Daplasca, Sabadell, Spain), PA (Nylon 6.6, type 200, DuPont) and PAN fibers (type 75, DuPont) from woven fabrics were used (Figure 1). Organics and metal salts (acetone, 4-np, NaOH, HCl, NaBH_4_, HNO_3,_ and AgNO_3_) from Panreac Company (Castellar del Vallès, Barcelona, Spain) were used as received.

### Pretreatment of the PUFs

The pretreatment of PUFs was investigated to activate the material. First, foams were washed with acetone and then with distilled water to eliminate the possible commercial treatments applied to the material. Different pretreatments were applied to 1 cm^3^ of foam samples, which were immersed in 25 ml of the pretreatment reagent solution (1 M HNO_3_, 3 M HNO_3_, 1 M NaOH, and 3 M NaOH) for 2 h under agitation. Afterwards, the samples were washed several times with distilled water.

In order to determine the possible effect of the pretreatments in the chemical structure of the PUFs, attenuated total reflectance Fourier transform infrared (FTIR-ATR) spectra were recorded with a Perkin Elmer Spectrum GX spectrometer (Norwalk, CT, USA). Moreover, for determining the concentration of the functional groups before and after the pretreatment of the matrix, two titration methods were applied to calculate IEC (in meq/g) of the material [16]:

1 For determining cation exchange groups: 1 cm^3^ of PUF was immersed in 100 ml of NaOH 0.1 M and shaked at room temperature for 48 h, time enough to ensure a complete neutralization of the acidic groups. Then, an aliquot of 10 ml was titrated with standardized HCl 0.1 M (3 replicates).

2 For determining anion exchange groups a similar procedure was used, but immersing the sample in 100 ml of HCl 0.1 M, and using standardized NaOH 0.1 M to titrate the 3 aliquots of 10ml.

### Synthesis of AgNPs

The synthesis of AgNPs in the polymeric matrices by the IMS methodology consisted of the following: (1) loading of the material with the metal ions (AgNO_3_ 0.4 M solution) and (2) reduction of metal ions to zero-valent MNPs through reaction (by using NaBH_4_ 0.5M solution). The reactions involved are as follows:

(1) $${\mathrm{R - X}}^{-}{\mathrm{Na}}^{+}{+\mathrm{Ag}}^{+}⇄\left({\mathrm{R - X}}^{-}\right){\mathrm{Na}}^{+}{+\mathrm{Ag}}^{+}$$

(2) $$\begin{array}{l} \left({\mathrm{R - X}}^{-}\right){\mathrm{Ag}}^{+}+\;{\mathrm{NaBH}}_{4}\\ \;+{3H}_{2}O\to \left({\mathrm{R - X}}^{-}\right){\mathrm{Na}}^{+}+{7/2\;H}_{2}+B{\left(\mathrm{OH}\right)}_{3}\\ \;+{\mathrm{Ag}}^{0} \end{array}$$

Although equations depict a pure ion exchange mechanism, the generation of coordination bonds between species may also result in the immobilization of the ionic species in the polymeric matrix. In addition, the entry of metal ions into the matrix could be significantly affected by the synthetic conditions (i.e., temperature) which can affect the structural organization of the polymer matrices thus making the matrix temporarily accessible to the metal ions by opening their structure; after the synthesis, the fibers revert back to their closely packed state thus trapping the MNPs within the polymer structure. For the PUFs, the procedure described above was performed at room temperature; whereas in the case of the textile fibers, synthesis using different temperatures (25°C, 40°C, and 80°C) were applied.

### Nanocomposite characterization

In order to determine the exact metal content in the prepared nanocomposites, samples of known weight were digested with concentrated HNO_3_. The resulting solutions (two replicates) were diluted and analyzed by inductively coupled plasma mass spectrometry (ICP-MS).

With the aim of characterizing the size and structure of the obtained AgNPs, transmission electron microscopy (TEM) was performed by a JEOL JEM-2011 HR-TEM (JEOL Ltd., Tokyo, Japan). Before observation, the samples were deposited between two plastic sheets in an epoxy resin, and ultra-thin slices were obtained using an ultra-microtome.

### Catalytic properties evaluation

The catalytic performance of nanocomposites was evaluated by using the reduction of 4-np to 4-ap by NaBH_4_ as a model reaction, which was considered to follow a pseudo-first-order kinetics, and the apparent rate constant (*k*_app_) was calculated. In a typical run, a piece of nanocomposite (1 cm^2^ for textile fibers and 1 cm^3^ for PUFs) was added to a vessel of 50 ml solution containing 4-np (0.5 mM) and NaBH_4_ (500 mM). The process was monitored at 390 nm by a Pharmacia LKB Novaspec II spectrometer (Biochrom Ltd., Cambridge, UK).

## Results and discussion

### Characterization of the polyurethane foams and their pretreatments

**Figure 2:**
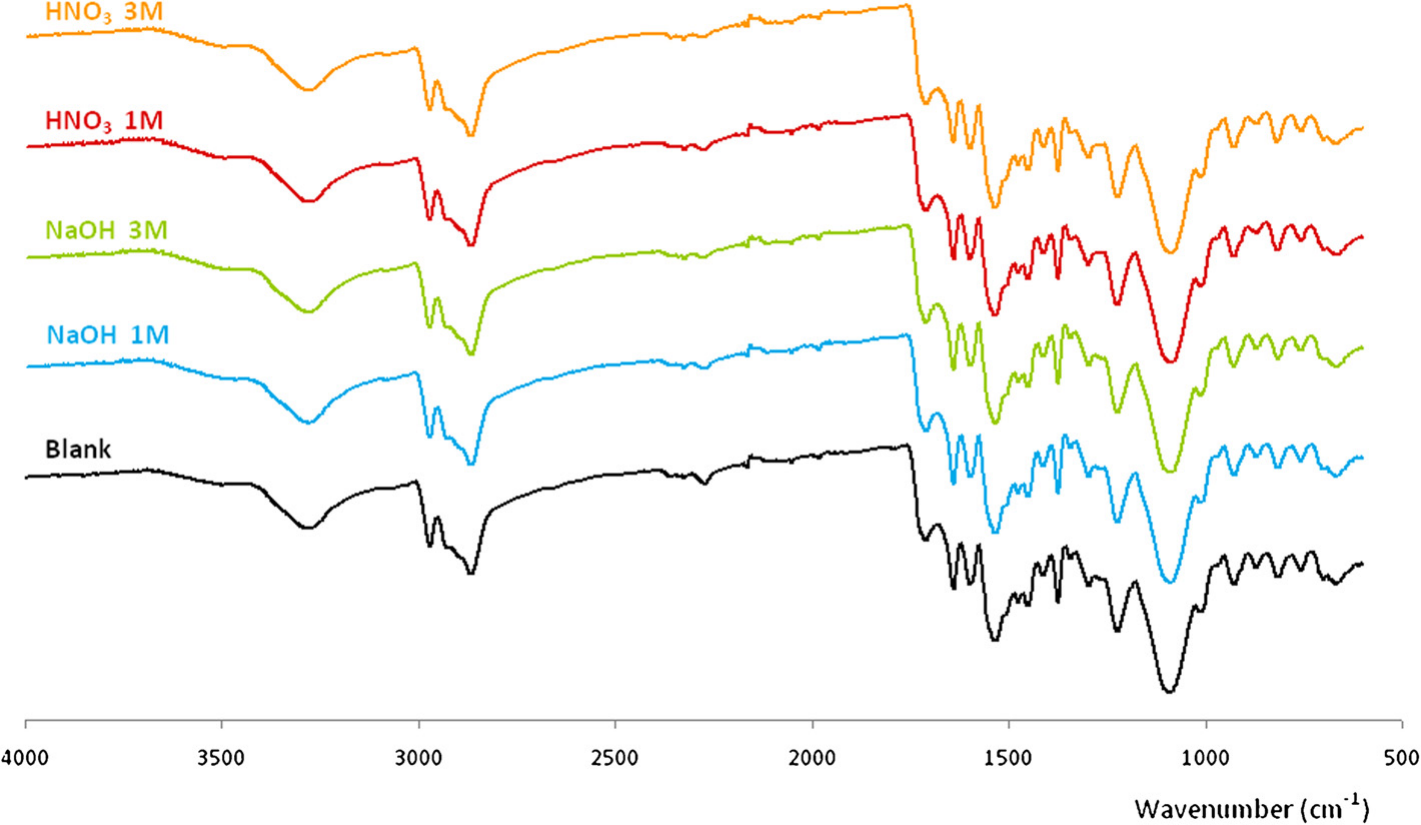
FTIR-ATR of PUFs before and after pretreatments.

PUF resulted to be a very stable material. The FTIR-ATR spectra of PUFs (Figure 2) show the distinctive polyurethrane (PU) bands [17]: the broad peak at 3,270 cm^−1^ is characteristic of the υ(N-H), the peaks at 1,690 and 1,520 cm^−1^ are typical for υ(C=O) (urethane band) and δ(NH) with υ(CO-N) (amide II). Surprisingly, no differences between spectra were observed. Thus, no chemical modification took place after any pretreatment. In addition, as seen in Table 1, similar values of IEC were obtained in all the cases, which also pointed out that a basic or acid pretreatment did not significantly affect the presence of ion-exchangeable positions.

**Table 1:** PUF IEC values

	**IEC (meq/g)**	
Treatment	Acid groups	Basic groups
Blank	0.65	0.62
NaOH 1M	0.32	0.61
NaOH 3M	0.57	0.61
HNO3 1M	0.66	0.71
HNO3 3M	0.61	0.57

IEC, ion exchange capacity. Uncertainty in all of the cases was <1%.

### Nanocomposites characterization

After applying the IMS technique, a darkening of the matrices was observed, indicative of the metal loading. The color for modified PUFs was similar, but clear differences in color intensity were detected for textile fibers: the higher the temperature, the darker the color.

**Figure 3:**
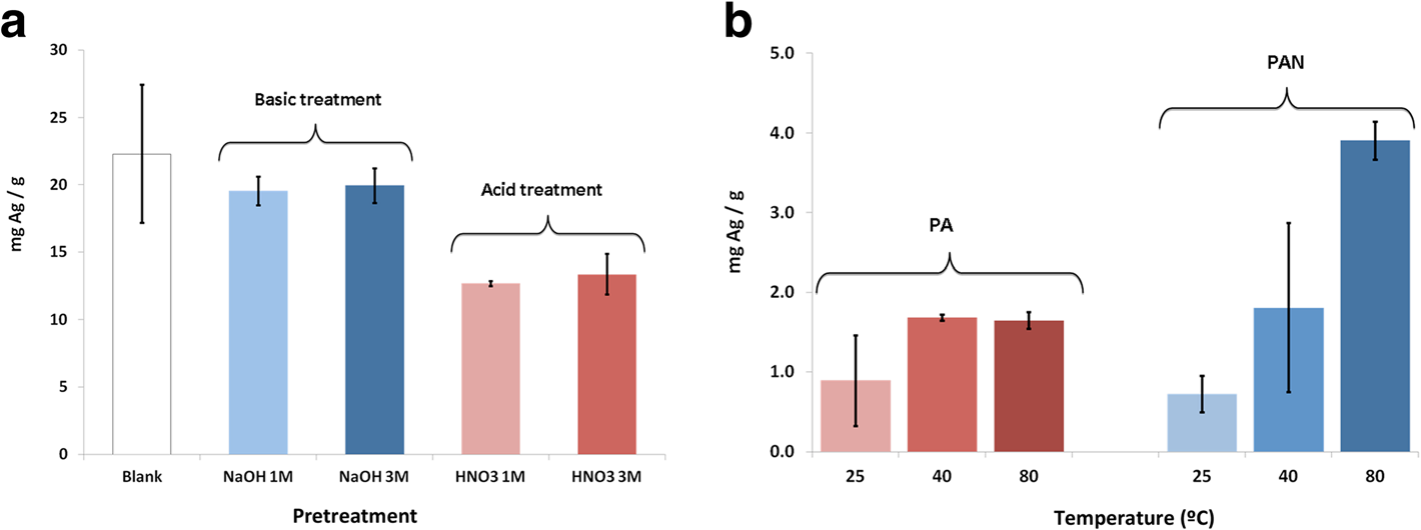
Results of the ICP-MS analysis of the Ag content in (a) PUFs and (b) PAN and PA fibers.

For PUFs, the metal content did not increase after pretreatments. On the one hand, a basic pretreatment allowed loading of metal in a similar way compared to untreated foams, whereas acid treatments resulted in a lower metal concentration (Figure 3). *A priori*, both treatments were expected to increase the total metal loading due to the formation of ionogenic groups. However, since no new ionogenic groups were generated (as concluded from the FTIR-ATR and from the IEC values), the loading of the Ag^+^ can be attributed to coordination with lone electron pairs of nitrogen atoms. Accordingly, the acid/basic treatments just ‘tune’ the possibility of coordination bonds to happen (depending on the isoelectric point of the matrix).

Very differently, for PAN fibers, increasing the temperature of the synthesis provided a higher metal loading. For PA fibers obtained at 40°C and 80°C, the metal content remains almost constant. In both cases, this can be explained because rising the temperature to the glass transition point of each polymer (*T*_g PAN_ = 85°C whereas *T*_g PA_ = 55°C) increases the macromolecular mobility of the glassy amorphous phase, enhancing the accessibility of the polymer matrix. This change is more notable in PAN fibers than in PA fibers due to the higher thermosensitivity of the mesomorphic PAN fibers [18] at temperatures around *T*_g_ in comparison with the more stable and high crystalline structure of the PA fibers. Basically, PAN fibers are strongly influenced by temperature because their structural organization is intermediate between amorphous and crystalline phases, whereas the strong intermolecular hydrogen bonds through the amide groups in PA fibers configure a more stable semi-crystalline structure which hinders the ion diffusion.

**Figure 4:**
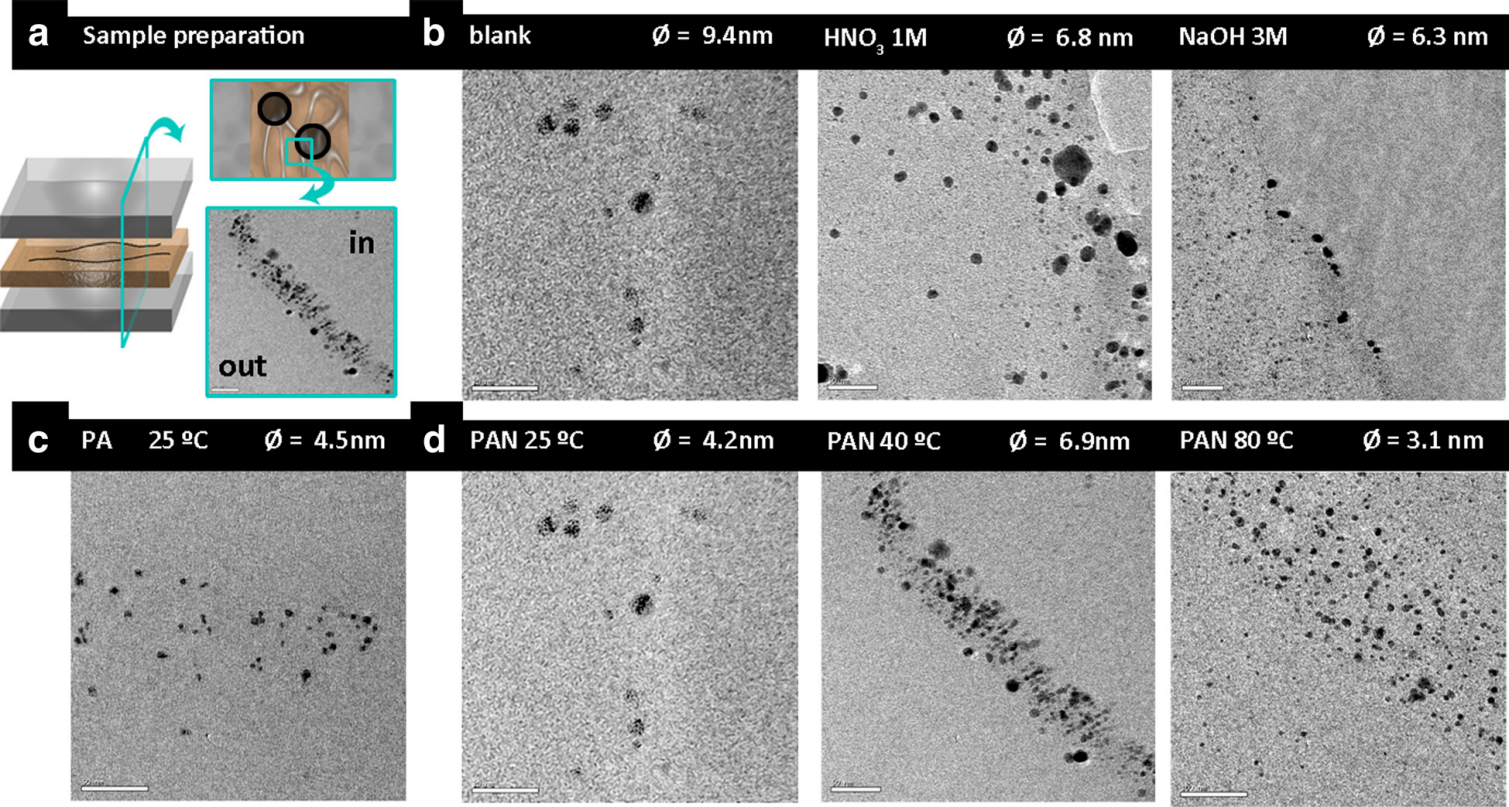
**TEM images of some matrices.** (**a**) Preparation of the ultra-thin films samples by cross-section for TEM analysis. TEM images obtained of (**b**) PUFs, (**c**) PA and (**d**) PAN fibers at different temperatures.

TEM images of some matrices are shown in Figure 4. Nanocomposites based on untreated PUFs showed large AgNPs on the surface, while smaller ones were observed inside the matrix. By applying any pretreatment, smaller AgNPs are obtained. When comparing PA (25°C) and PAN (25°C), it was observed that there was a higher content of AgNPs for PA, but all the MNPs showed similar diameters. Yet, more MNPs were found for samples synthesized at higher temperatures, very probably because a higher diffusion of the AgNPs inside the matrix was achieved. The MNPs average diameter (Ø) was determined by counting between 200 and 300 MNPs per sample, representing the corresponding size distribution histograms that were fitted to a Gaussian curve of the three parameters [10].

### Catalytic evaluation

**Figure 5:**
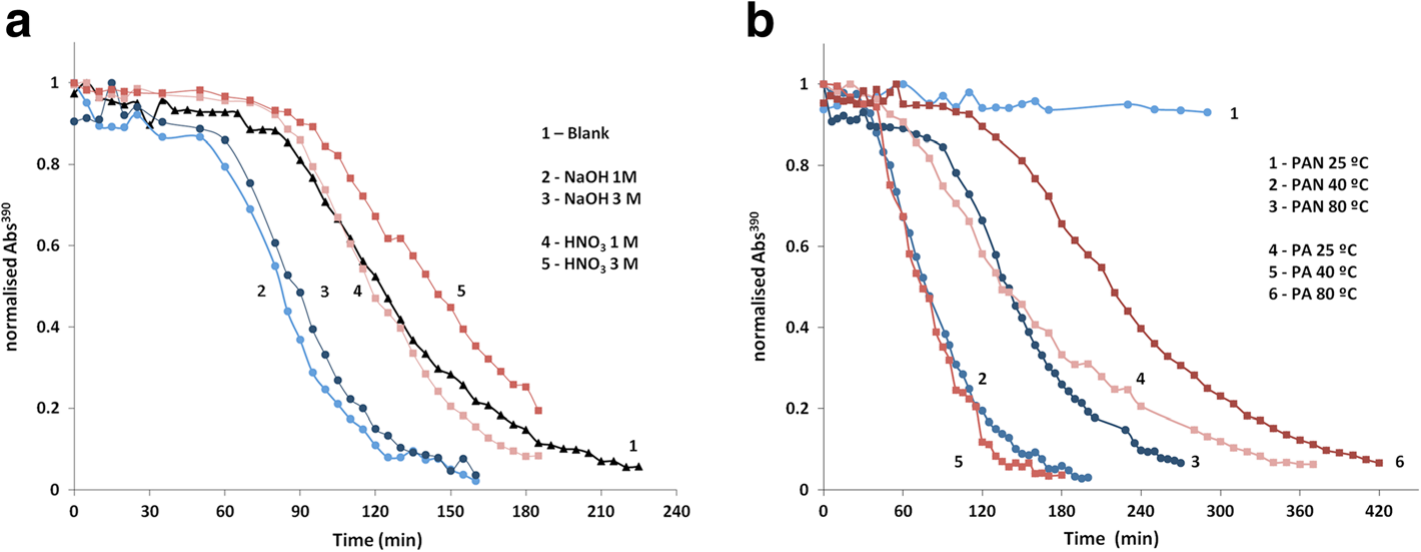
Catalytic evaluation of (a) PAN and PA nanocomposite fibers and (b) PUFs nanocomposites.

Only PUFs and textile fibers containing AgNPs exhibited catalytic activity when evaluated in batch tests (Figure 5). The only nanocomposite without catalytic activity was PAN (25°C), which also contains the lowest amount of AgNPs. Reaction rate values (Table 2) increased for the PUFs with basic pretreatments. However, in PUFs with HNO_3_ pretreatments, even if their metal content was lower (*c.a.* 40% less), the normalized catalytic activity remained almost constant. This fact can be explained because of the smaller AgNPs diameters obtained with the pretreatments which implies a higher catalytic area for the same amount of metal.

**Table 2:** **Reaction rates (k**_**app**_**) obtained for each nanocomposite**

	**Pretreatment / T (°C)**	***k***_**app **_**(s**^**−1**^**·mg**_**Ag**_^**−1**^**)**
PUFs	Blank	0.05
	NaOH 1M	0.10
	NaOH 3M	0.10
	HNO_3_ 1M	0.12
	HNO_3_ 3M	0.06
PAN	25°C	-
	40°C	0.47
	80°C	0.13
PA	25°C	0.49
	40°C	0.40
	80°C	0.31

For textile fibers (except PAN prepared at 25°C), increasing the temperature of synthesis decreased the reaction rate. For PAN fibers, this can be clearly explained by TEM images: at a higher temperature, some of the AgNPs were formed inside the matrix and, therefore, they might not be accessible to the reagents.

**Figure 6:**
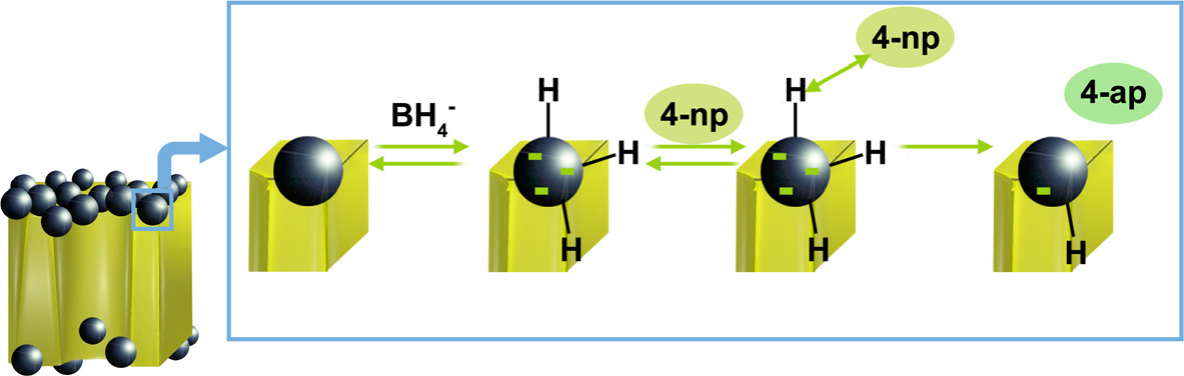
**Langmuir-Hinselwood mechanism for the reduction of 4-np to 4-ap with NaBH**_**4**_**.**

Although almost all of the nanocomposites exhibited good catalytic activity for the reduction of 4-np, an induction time was needed for the reaction to proceed at high extent. This induction time has also been observed in other works for PdNPs [9,11,19,20], where it has usually been suggested that H_2_ evolved from the decomposition of NaBH_4_ can be loaded inside PdNPs competing with the catalytic reaction. Thus, once the absorption of H_2_ has reached a saturation value, the catalytic reaction prevails. As far as we know, in the case of silver, this situation has not been already described but is very compatible with the experimental results. In fact, taking into account the well-known and fully accepted Langmuir-Hinshelwood mechanism for the reduction of 4-np to 4-ap [19], there is a first step during the reaction that involves the loading of the catalytic nanoparticles with hydride (H^−^). Figure 6 illustrates the aforementioned mechanism.

## Conclusions

The synthesis AgNPs in PUFs and textile fibers was successfully achieved: small nonaggregate MNPs were obtained in all of the matrices and mainly located on the surface. Neither acid nor basic pretreatments significantly affected the metal loading in PUFs. Instead, a tuning effect of the matrix after applying different pretreatments was observed, since the AgNPs distribution and size depended on the treatment. For textile fibers, the higher the temperature of synthesis, the higher the metal loading, very probably due to macromolecular chains mobility. In addition, for PAN fibers, the temperature significantly affected the spatial distribution of AgNPs due to the low values of the glass transition temperature. Almost all of the nanocomposites exhibited good catalytic activity for the reduction of 4-np, although an induction time was needed for the reaction to proceed at high extent. From these results, it comes that catalytic efficiency not only depends on the metal loading but also on the MNPs’ diameter and their spatial distribution. Finally, these results prove that matrices not bearing ion-exchangeable groups can also be successfully used for nanocomposites synthesis by IMS.

## Competing interests

The authors declare that they have no competing interests.

## Authors’ contributions

KZ carried out the experimental part concerning the polyurethane foams characterization, nanocomposite synthesis and characterization, and their catalytic evaluation. BD participated in the design and coordination of the study, carried out the experimental part concerning the textile fibers characterization, nanocomposite synthesis and characterization, catalytic evaluation, and wrote the main part of the manuscript. JM conceived the study and participated in its design and coordination. FC participated in the experimental design and interpretation of the textile fibers nanocomposites procedure and results. MM and DNM participated in the interpretation of the results. All authors read and approved the final manuscript.
